# Three Proteins (Hpa2, HrpF and XopN) Are Concomitant Type III Translocators in Bacterial Blight Pathogen of Rice

**DOI:** 10.3389/fmicb.2020.01601

**Published:** 2020-07-22

**Authors:** Xuyan Mo, Liyuan Zhang, Yan Liu, Xuan Wang, Jiaqi Bai, Kai Lu, Shenshen Zou, Hansong Dong, Lei Chen

**Affiliations:** ^1^College of Plant Protection, Nanjing Agricultural University, Nanjing, China; ^2^College of Plant Protection, Shandong Agricultural University, Tai’an, China; ^3^Crop Molecular Biology Research Group, State Key Laboratory of Crop Biology, Tai’an, China

**Keywords:** *Xanthomonas oryzae* pv. *oryzae*, PthXo1, type III translocator, Hpa2, HrpF, XopN

## Abstract

Type III (T3) proteic effectors occupy most of the virulence determinants in eukaryote-pathogenic Gram-negative bacteria. During infection, bacteria may deploy a nanomachinery called translocon to deliver T3 effectors into host cells, wherein the effectors fulfill their pathological functions. T3 translocon is hypothetically assembled by bacterial translocators, which have been identified as one hydrophilic and two hydrophobic proteins in animal-pathogenic bacteria but remain unclear in plant pathogens. Now we characterize Hpa2, HrpF, and XopN proteins as concomitant T3 translocators in rice bacterial blight pathogen by analyzing pathological consequences of single, double, and triple gene knockout or genetic complementation. Based on these genetic analyses, Hpa2, HrpF, and XopN accordingly contribute to 46.9, 60.3, and 69.8% proportions of bacterial virulence on a susceptible rice variety. Virulence performances of Hpa2, HrpF, and XopN were attributed to their functions in essentially mediating from-bacteria-into-rice-cell translocation of PthXo1, the bacterial T3 effector characteristic of transcription factors targeting plant genes. On average, 61, 62, and 71% of PthXo1 translocation are provided correspondingly by Hpa2, HrpF, and XopN, while they cooperate to support PthXo1 translocation at a greater-than-95% extent. As a result, rice disease-susceptibility gene *SWEET11*, which is the regulatory target of PthXo1, is activated to confer bacterial virulence and induce the leaf blight disease in rice. Furthermore, the three translocators also undergo translocation, but only XopN is highly translocated to suppress rice defense responses, suggesting that different components of a T3 translocon deploy distinct virulence mechanisms in addition to the common function in mediating bacterial effector translocation.

## Introduction

Many pathogenicity-determinative proteic effectors of Gram-negative bacteria, which are plant (Alfano and Collmer, 2004; Nissinen et al., 2007; White et al., 2009), or animal (Chatterjee et al., 2013; Domingues et al., 2016; Piscatelli et al., 2016) pathogens, are secreted by the bacterial type III (T3) secretion system. Subsequently, effectors must be translocated from bacterial cells into eukaryotic host cells in order to fulfill a virulent role in susceptible hosts and an avirulent role in resistant hosts (Bogdanove and Voytas, 2011; Zhang et al., 2015; Chen et al., 2017). T3 effector translocation follows the translocon-dependent or -independent model depending on variations in the genetic repertoire of bacterial T3 translocators (Julie and Guy, 2016; Shanmugam and Dalbey, 2019; Zhang et al., 2019a). The translocon-independent model emphasizes endocytosis of effectors through direct interaction with recognizing compounds situated in plasma membranes (PMs) of eukaryotic cells (Rüter et al., 2010; Santi-Rocca and Blanchard, 2017). The translocon-dependent model is conceived as a nanomachinery called “T3 translocon” (Büttner and Bonas, 2002), which is hypothetically formed by interactions of bacterial T3 translocators with each other and with specific recognizing constituents (Ji and Dong, 2015b; Büttner, 2016), both lipids (Büttner et al., 2002; Haapalainen et al., 2011), and proteins (Oh and Beer, 2007; Li et al., 2015, 2019), associated with eukaryotic PMs. In the translocon-dependent model, recognition of the hydrophilic translocator by a PM constituent is the first step toward the translocon assembly (Goure et al., 2004; Mueller et al., 2008). Lipid binding of hydrophobic translocators finalize the translocon with an inner channel to accommodate effector translocation (Mueller et al., 2008; Büttner, 2012; Ji and Dong, 2015b; Zhang et al., 2019a).

In relevance, a big problem is the paucity in knowledge about the T3-translocon composition or the number of T3 translocators that plant-pathogenic bacteria must possess (Ji and Dong, 2015a; Scheibner et al., 2017; Zhang et al., 2019b). It has been determined that T3 translocators of animal-pathogenic bacteria include one hydrophilic protein and two hydrophobic proteins (Ji and Dong, 2015b; Büttner, 2016). Whether a similar repertoire exists in plant-pathogenic bacteria remains unknown. Plant-pathogenic bacteria occupy more than 100 species in the current classification, but only 5 species have been studied in terms of T3 translocators. The first identified T3 translocator was the atypical hydrophobic protein HrpF in *Xanthomonas campestris* pv. *vesicatoria* (*Xcv*), the bacterial spot pathogen of pepper (Büttner et al., 2002; Noël et al., 2002). Then, HrpF homologs designated as PopF1 and PopF2 were shown to be concomitant translocators of the transcription activator-like effectors (TALE) AvrA in *Ralstonia solanacearum*, the pathogen that causes bacterial wilt in a variety of plant species (Meyer et al., 2006). The same pathogen has a different T3-translocator called PopA (Racape et al., 2005), one of harpin-group hydrophilic proteins that function as T3 accessory components (Choi et al., 2013; Ji and Dong, 2015b). Later, T3 translocators were characterized as four harpins (HrpK1, HrpZ1, HrpW1, and HopAK1) in *Pseudomonas syringae* pv. *tomato* (*Pst*), which causes bacterial speck in a variety of plant species (Kvitko et al., 2007), and the harpin HrpN_Ea_ in *Erwinia amylovora*, the bacterial pathogen that causes fire blight in rosaceous plants (Bocsanczy et al., 2017, 2008). Subsequently, HrpF and the harpin protein Hpa2 were found to be concomitantly required for T3 effector translocation from the rice bacterial stripe pathogen *X. oryzae* pv. *oryzicola* into cells of the host plant and the non-host tobacco (Li et al., 2011). To date, however, none of plant-pathogenic bacteria has been characterized with respect to the T3 translocon composition. In other words, it is unclear at present how many T3 translocators must be produced to ensure the translocation of T3 effectors by an infecting community (species, subspecies, or pathovar) of plant-pathogenic bacteria.

We have extensively studied pathological and physiological functions of the harpin protein Hpa1 from the rice bacterial pathogen *Xanthomonas oryzae* (Peng et al., 2004; Liu et al., 2006; Chen et al., 2008a, b; Sang et al., 2012; Li et al., 2013, 2014, 2015, 2019; Ji and Dong, 2015a, b; Dong et al., 2016; Wang et al., 2018; Zhang et al., 2019a). We previously characterized Hpa1 as a hydrophilic protein (Chen et al., 2008a) and recently demonstrated its role in T3 effector translocation from *X. oryzae* pv. *oryzae* (*Xoo*), the pathogen that causes bacterial blight in rice, into the host plant cells (Wang et al., 2018). Like all bacteria in the *Xanthomonas* genus, *Xoo* causes disease by the virulence role of T3 effectors, either TALEs, including Avr and Pth proteins, or non-TALEs, mainly *Xanthomonas* outer proteins (Xops) as nominated with the bacterial genus landmark (White et al., 2009; Bogdanove and Voytas, 2011). In general, TALEs and non-TALEs are secreted along with harpins by the T3 system in a chronological pattern (Roden et al., 2004; Wang et al., 2018) and then translocated into plant cells to play a virulent or avirulent role depending on plant varieties (Büttner, 2016). Moreover, *Xoo* is the most devastating bacterial pathogen of rice in China and other parts of East Asia, Southeast-Asian countries like Philippine, United States and other parts of North America, Oceanian countries including Australia, and West Africa as well (Mew, 1987). Also, *Xoo* is a model of plant bacterial pathogen used by the plant pathology community (Niño-Liu et al., 2006; Mansfield et al., 2012).

We use this bacterial model to determine the T3 translocator composition in plant bacterial pathogen. The major attempt is to know whether analogs of both hydrophobic and hydrophilic translocators already identified in animal-pathogenic bacteria (Büttner, 2012; Ji and Dong, 2015b) are required for plant-pathogenic bacteria to translocate their T3 effectors. By such studies, we will be able to judge how many translocators are required for the translocation of a T3-effector from a species of plant bacterial pathogen or a pathovar of the bacterial species. We have demonstrated that the function of Hpa1 as a T3 translocator essentially contributes to virulence of the *Xoo* strain PXO99^A^ in the susceptible *japonica* rice variety Nipponbare (Wang et al., 2018). Another pivotal determinant of PXO99^A^ virulence is the TALE PthXo1 (Yang et al., 2006). In Nipponbare, PthXo1 supports the bacterial virulence by activating the host susceptibility gene, *OsSWEET11*, synonym *Os8N3* (Yang et al., 2006), which encodes a sugar transporter protein (Chen et al., 2010). Thus, the production and translocation of PthXo1 has the function of supporting sugar secretion from rice cells to provide potential nutrition for bacterial multiplication in the apoplastic space (Chen et al., 2010). In PXO99^A^-infected Nipponbare plants, Hpa1 serves as a translocator for PthXo1 and this function is indispensable for PthXo1 to activate *OsSWEET11* expression (Wang et al., 2018).

The present study was devised to assess the possible role of Hpa2 and HrpF in PthXo1 translocation from PXO99^A^ bacteria into rice cells because both proteins were previously identified as T3 translocators in other plant-pathogenic bacteria (Büttner et al., 2002; Li et al., 2011; Scheibner et al., 2017). Meanwhile, XopN was also considered due to its structural and functional characteristics. XopN is a pluripotent effector present in different *Xanthomonas* species, has the structural features of molecular adaptors, and therefore confers virulence by interacting with immunity-relevant proteins in plants (Roden et al., 2004; Taylor et al., 2012). After secreted by *Xcv* in infecting tomato, XopN localizes to the PM-cytoplasm interface, whereon XopN interacts with the plant receptor-like protein kinase TARK1 (Kim et al., 2009), and 14-3-3 proteins (Taylor et al., 2012) that also function as molecular adaptors (Camoni et al., 2018). As the PM localization is a common characteristic of T3 translocators (Mueller et al., 2008; Ji and Dong, 2015b; Zhang et al., 2019a), XopN is involved in the present study. We present evidence that the three proteins (Hpa2, HrpF, and XopN) secreted by PXO99^A^ in infecting Nipponbare plants contribute to different proportions of PthXo1 translocation. We further show that XopN executes an additional function, inducing marked suppression of rice defense responses following a substantial translocation into rice cells.

## Results

### Hpa2, HrpF, and XopN Synergistically Confer PXO99^A^-Bacterial Virulence

**FIGURE 1:**
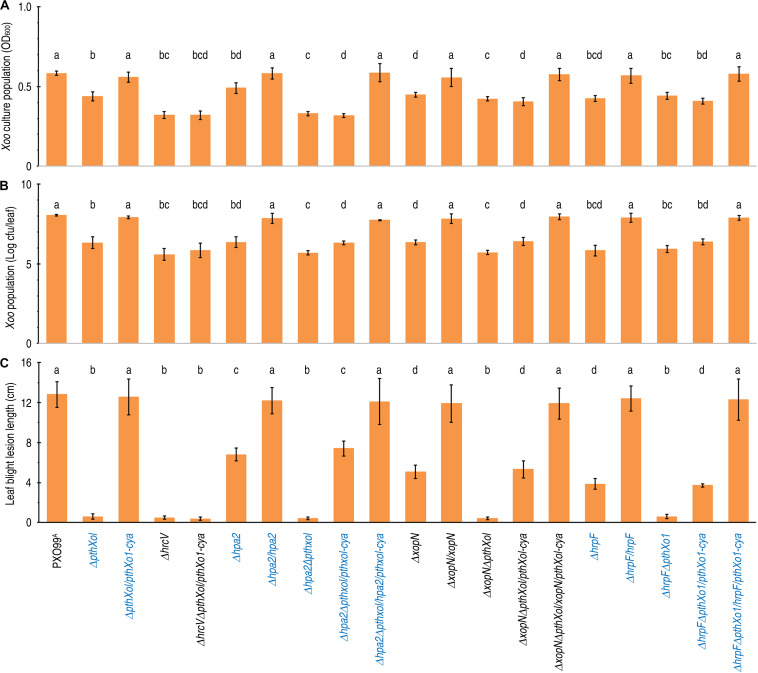
The effects of *hpa1*, *hpa2*, *hrpF*, and *hrcV* on *Xoo* bacterial multiplication in culture and virulence on the susceptible rice variety Nipponbare. **(A–C)** The *Xoo* WT strain PXO99^A^ and its mutants generated by deleting the corresponding genes were tested in parallel experiments. Data shown are mean values ± standard deviation (SD) estimates. Number of biological repeats (*n*) = 6 independent experiments in **(A)**; *n* = 30 inoculated leaves from 15 plants tested in 3 independent experiments in **(B)** and **(C)**. Different letters on bar graphs indicate significant differences of data obtained from the experiments performed on the different bacterial strains (ANOVA and Duncan’s test, *P* < 0.001 in **(A)** and **(C)**, *P* < 0.005 in **B**). **(A)** Bacterial populations of the different *Xoo* strains in a liquid medium after 20-h incubation. **(B)** Populations of bacteria multiplied in leaves. Leaves of 30-day-old Nipponbare rice seedlings were inoculated by leaf-top clipping with clinical scissors immediately dipped in the corresponding bacterial suspensions of the *Xoo* strains. Three days later, bacterial population in leaf tissues were determined. **(C)** Lesion length of bacterial blight on leaves 12 dpi.

In order to assess the individual roles of Hpa2, HrpF, and XopN in PXO99^A^-bacterial virulence on Nipponbare plants, virulence levels were compared when the *hpa2*, *hrpF*, and *xopN* genes were present canonically in the PXO99^A^ genome, individually deleted from and then backfilled to the genome. Involved experimental operations were performed by conventional procedures (Yang et al., 2006; Wang et al., 2018). Gene knockout by double-crossover homologous recombination (Song and Yang, 2010) created the bacterial mutants Δ*Hpa2*, Δ*HrpF*, and Δ*XopN*, respectively, while bringing the wild-type (WT) single genes back to the corresponding mutants resulted in the generation of genetically complementing strains Δ*hpa2/hpa2*, Δ*hrpF/hrpF*, and Δ*xopN/xopN* (Supplementary Table 1). To assess the effects of Hpa2, HrpF, and XopN on bacterial multiplication, the different bacterial strains were subjected to 20-h culture in nutrient broth (NA) liquid medium. The gene-knockout mutants Δ*hpa2*, Δ*hrpF*, and Δ*xopN* showed to be significantly (*P* < 0.001) inferior to the WT stain in bacterial populations, whereas, genetic complementation restored these mutants to the WT in extents of multiplication in the medium (Figure 1A). Evidently, Hpa2, HrpF, and XopN are necessary for full multiplication of bacteria under the culturing condition. Moreover, Hpa2, HrpF, and XopN provided substantial contributions to bacterial virulence, which was evaluated by using two recognized criteria: populations of antibiotics-labeled bacteria (Supplementary Table 1) propagated within inoculated leaves of the plants and severities of blight symptoms subsequently formed on the leaves.

**FIGURE 2:**
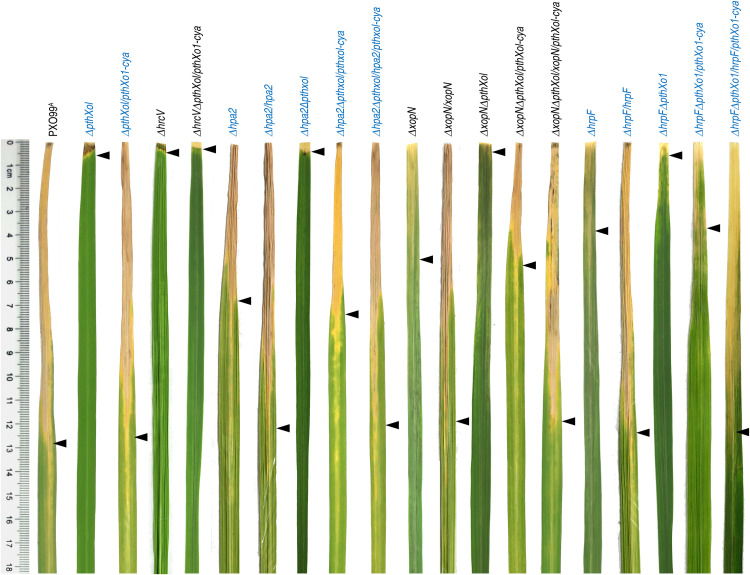
Bacterial blight symptoms on leaves of rice plants inoculated separately with PXO99^A^ and mutants related to *hpa1*, *hpa2*, *hrpF*, and *hrcV*. Inoculation was performed on 30-day-old Nipponbare rice seedlings by using the leaf-clipping method. 12 h later, inoculated leaves were excised, spread on papers by the aid of sellotapes, and photographed. Leaf images were aligned, brightness/contrast was adjusted by a uniform extent, and background shadings were eliminated by using the magic wand and cut-off tools in Photoshop. Each leaf photo represents 30 leaves of 15 plants assessed in 3 independent experiment.

To setup bacterial virulence assays, two expanded leaves of 30-day-old Nipponbare seedlings were inoculated by leaf-top clipping with clinical scissors immediately dipped in a bacterial suspension of the bacterial WT and recombinant strains. We first compared the WT strain PXO99^A^, single-gene-knockout mutants (Δ*hpa2*, Δ*hrpF*, and Δ*xopN*), and subsequently complemented strains (Δ*hpa2/hpa2*, Δ*hrpF/hrpF*, and Δ*xopN/xopN*) in terms of the two criteria for virulence assessment. When determined at the 3rd day post-inoculation (dpi), the bacterial WT and complementing strains displayed regular multiplication in leaf tissues, gaining bacterial populations around 10^8^ colony formation unit (cfu) per leaf, but the Δ*hpa2*, Δ*hrpF*, and Δ*xopN* mutants incurred significant reductions in cfu/leaf values (Figure 1B). In the subsequent 10 days, the blight symptoms developed in inoculated leaves with great variations in the visible severities as caused by the different bacterial genotypes, in which the Δ*hpa2*, Δ*hrpF*, and Δ*xopN* mutants were much less aggressive than the WT and complementing strains (Figure 2). This difference was confirmed by digitizing the disease severity as leaf blight lesion length, which was significantly (*P* < 0.005) shorter in leaves inoculated with the bacterial mutant Δ*hpa2*, Δ*hrpF*, or Δ*xopN* in contrast to the WT and complementing strains (Figure 1B). On average, 46.9, 60.3, and 69.8% reductions in leaf blight lesion length were caused by the corresponding gene deletion of Δ*hpa2*, Δ*hrpF*, and Δ*xopN* from the PXP99^A^ genome. These data suggest that Hpa2, HrpF, and XopN are integral virulence components of PXO99^A^, contributing to different proportions of PXO99^A^-bacterial virulence on the susceptible rice variety.

### None of Hpa2, HrpF, and XopN Affects PthXo1 Secretion

**FIGURE 3:**
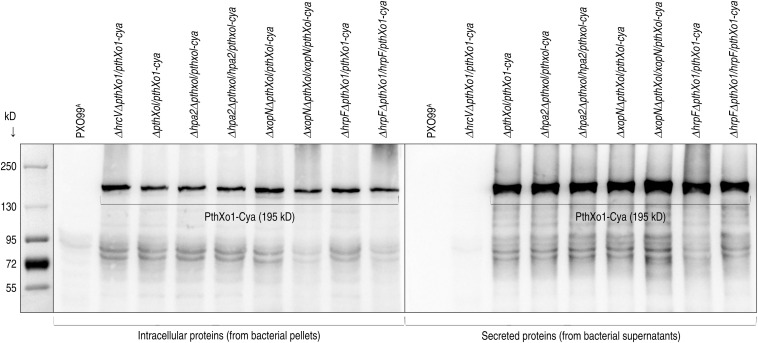
PthXo1 secretion by PXO99^A^ and mutants defected in *hpa1*, *hpa2*, *hrpF*, or *hrcV*. The PthXo1 protein was fused to a Cya sequence and introduced in the fusion form into bacteria of the PXO99^A^ mutants. Bacterial cultures of PXO99^A^ and the mutants were used to isolate soluble proteins. Protein samples were analyzed by immunoblotting with the specific antibody against Cya.

We looked for functional connections of Hpa2, HrpF, and XopN, which differentially contribute to PXO99^A^ virulence on the rice variety Nipponbare (Figures 1, 2), with the bacterial TALE PthXo1, which determines the bacterial virulence (Yang et al., 2006) following secretion by the bacterial T3 pathway and translocation into Nipponbare cells (Li et al., 2019). Thus, we analyzed the effects of Hpa2, HrpF, and XopN on PthXo1 secretion and translocation. Both types of the molecular trafficking were analyzed by using calmodulin-dependent adenylate cyclase (Cya), a widely employed bacterial secretion reporter under culture conditions and a eukaryotic cytoplasmic import marker in plant and animal tests (Chakravarthy et al., 2017). We verified that the fused Cya did not affect the virulent role of PthXo1 in Nipponbare plants following inoculation by the leaf-top clipping with a bacterial suspension of the recombinant bacterial strain Δ*pthXo1/pthXo1-cya* in contrast to the WT strain PXO99^A^. Instead, Δ*pthXo1/pthXo1-cya* resembled PXO99^A^ in multiplication within inoculated leaf tissues (Figure 1B) to cause leaf blight (Figure 2) with an equivalent severity shown as lesion length (Figure 1C). Immunoblotting analysis indicated that PthXo1-Cya was secreted by the bacterial T3 secretion pathway in the presence of HrcV (Figure 3), an inner membrane protein essential for substrate docking into the T3 system in *Xoo* (Sugio et al., 2005). In contrast to PthXo1, β-lactamase used as a lysis control was not secreted, by contrast, it remained inside bacterial cells no matter whether *hrcV* was present or absent (Figure 3), confirming the specificity in PthXo1 secretion by the T3 secretion pathway. Meanwhile, PthXo1-Cya was secreted equally well whether *hpa2*, *hrpF*, and *xopN* were deleted or remained in the PXO99^A^ genome, suggesting that none of Hpa2, HrpF, and XopN is required for the effector secretion.

### Hpa2, HrpF, and XopN Are Concomitant Translocators of PthXo1

**FIGURE 4:**
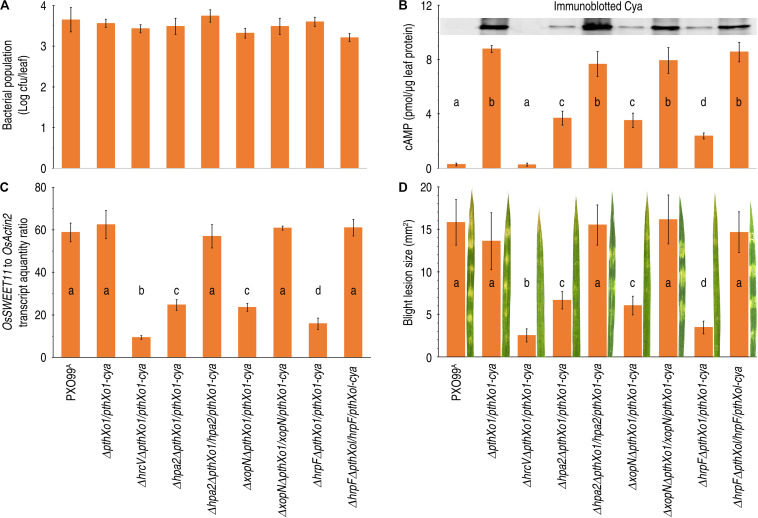
The effects of *hpa1*, *hpa2*, and *hrpF* on PthXo1 translocation and bacterial virulence. **(A–D)** Leaves of 14-day-old Nipponbare rice seedlings were inoculated by infiltration with bacterial suspensions of PXO99^A^ and mutants defected in *hpa1*, *hpa2*, and *hrpF*, respectively. Inoculated leaves were excised and used in the different assays. Quantitative data are given as the means ± SDs. Different letters on bar graphs indicate significant differences of data obtained from the experiments performed on the different bacterial strains (ANOVA and Duncan’s test, *P* < 0.001, *n* = 3 independent experiment each involving 30 leaves of 15 plants). **(A)** Bacterial population in leaves 12 hpi. **(B)** PthXo1-Cya translocation levels shown as cytoplasmic cAMP concentrations in leaves 12 hpi. Inset shows immunoblotting of leaf proteins hybridized in the blot with specific anti-Cya antibody. **(C)** Rice *OsSWEET11* gene expression in leaves 48 hpi. Gene expression was analyzed by RT-qPCR using the constitutively expressed *OsActin2* gene as a reference. **(D)** Leaf blight severities and leaf images assessed at 7 dpi.

In the Cya reporter assay using rice leaves inoculated by the leaf-center-infiltrating method as did usually (Kvitko et al., 2007; Chakravarthy et al., 2017; Li et al., 2019), Hpa2, HrpF, and XopN were found to partake in PthXo1 translocation from PXO99^A^-bacterial cells into the cytosol of Nipponbare cells. To initiate the assay, two elder leaves of 14-day-old Nipponbare seedlings were infiltrated with every bacterial suspension of pertinent *Xoo* strains. They were PXO99^A^, Δ*pthXo1/pthXo1-cya*, Δ*hrcVΔpthXo1/pthXo1-cya*, Δ*hpa2*Δ*pthXo1/pthXo1-cya*, Δ*hpa2ΔpthXo1/hpa2/pthXo1-cya*, Δ*xopNΔpthXo1/pthXo1-cya*, Δ*xopNΔpthXo1/xopN/pthXo1-cya*, Δ*hrpFΔpthXo1/pthXo1-cya*, and Δ*hrpF*Δ*pthXol/hrpF/pthXol-cya*. After 12 h post-inoculation (hpi), leaves were sampled and used in determinations of TAL effector translocation of the *in planta Xoo* populations (Figure 4A) and PthXo1 translocation (Figure 4B). At 12 hpi, bacterial populations of the different strains in leaves were similar regardless of *hpa1*, *hrpF*, or *xopN* deletion (Figure 4A). In this case, the possible effect of bacterial population on quantitative changes in PthXo1 translocation from the different bacterial strains could be excluded. PthXo1 translocation was verified by immunoblotting of soluble proteins isolated from inoculated rice leaves and hybridized with αCya, the specific anti-Cya antibody (Figure 4B, inset). The Cya reporter system allows for accurate quantification of an effector moved into eukaryotic cells based on the cytosolic concentration of cAMP as an exclusive product of the effector-Cya activity (Chakravarthy et al., 2017). Thus, levels of PthXo1-Cya translocation were quantified as cAMP concentrations during the effector-Cya activity in leaf cells (Figure 4B, bar graph). In both analyses, little translocation was found with Δ*hrcV/pthXo1-cya* due to the absence of HrcV-mediated secretion (Figure 4).

Immunoblotting clearly showed the αCya-hybridized protein bands indicating translocation of the PthXo1-Cya fusion protein from PXO99^A^ cells into the cytosol of Nipponbare rice cells (Figure 4B, inset). However, the αCya-hybridized protein bands were detected from the Nipponbare plants inoculated with the bacterial strains Δ*pthXo1/pthXo1-cya*, Δ*hpa2ΔpthXo1/hpa2/pthXo1-cya*, Δ*xopNΔpthXo1/xopN/pthXo1-cya*, and Δ*hrpF*Δ*pthXol/hrpF/pthXol-cya*, which contain *hpa2*, *hrpF*, and *xopN*, respectively. In the case of genetic modification that had deleted any of the three genes, as did in the bacterial strains Δ*hpa2*Δ*pthXo1/pthXo1-cya*, Δ*xopNΔpthXo1/pthXo1-cya*, or Δ*hrpFΔpthXo1/pthXo1-cya*, αCya-hybridized protein bands were not present in the protein blot (Figure 4B, inset). In the quantitative assay, high concentrations of cAMP were detected in leaves inoculated with the bacterial strains Δ*pthXo1/pthXo1-cya*, Δ*hpa2ΔpthXo1/hpa2/pthXo1-cya*, Δ*xopNΔpthXo1/xopN/pthXo1-cya*, and Δ*hrpF*Δ*pthXol/hrpF/pthXol-cya*, respectively. Cya activity was substantially decreased due to deletion of *hpa1*, *hrpF*, or *xopN*, as evidenced by a significant (*P* < 0.001) reduction in the cytoplasmic cAMP quantity in leaves inoculated with any of the Δ*hpa2*Δ*pthXo1/pthXo1-cya*, Δ*xopNΔpthXo1/pthXo1-cya*, and Δ*hrpFΔpthXo1/pthXo1-cya* strains (Figure 4B, bar graph). Both qualitive and quantitative analysis results demonstrate that Hpa2, HrpF, and XopN share the concomitant function in PthXo1 translocation from bacteria into rice cells. Comparing cAMP concentrations in rice leaves inoculated with the bacterial strain Δ*pthXo1/pthXo1-cya* and the specific gene-knockout strains, Hpa2, HrpF, and XopN occupy average 60.5, 62.2, and 71.2% proportions of PthXo1 translocation under the condition in this study.

### The Three Translocators Synergistically Promote PthXo1 to Perform Its Virulent Role

We assumed that the role of Hpa2, HrpF, and XopN in Pthxo1 translocation might cause *OsSWEET11*, the transcriptional target of PthXo1 (Yang et al., 2006), to be expressed in a manner of dependence on Hpa2, HrpF, and XopN present in the bacteria inoculated to rice plants. We examined this hypothesis by determining *OsSWEET11* transcript quantities in Nipponbare leaves following inoculation by leaf infiltration with every bacterial suspension of the proper *Xoo* strains (Figure 4). Analyses by RT-qPCR, namely real-time reverse transcriptase (RT) polymerase chain reaction (PCR), indicated that *OsSWEET11* was highly expressed in plants inoculated with the WT *Xoo* strain (PXO99^A^) or recombinant strains that concurrently contain *pthxo1*, *hpa2*, *hrpF*, and *xopN* (Figure 4C). RT-qPCR data also demonstrated that *OsSWEET11* expression was significantly (*P* < 0.001) decreased in Nipponbare leaves inoculated with bacteria in which any of *pthXo1*, *hpa2*, *hrpF*, and *xopN* had been delete. Consistently, scoring leaf blight lesion size confirmed that recombinant *Xoo* strains that concurrently contain *pthxo1*, *hpa2*, *hrpF*, and *xopN* showed to be as aggressive as the WT strain in causing leaf blight symptoms, but the bacterial aggression incurred significant (*P* < 0.001) impairments by deleting any of these genes from the bacterial genome (Figure 4D). These results highlight the mechanistic sequence of pathological responses from the concomitant function of three translocators (Hpa2, HrpF, and XopN) in PthXo1 translocation, PthXo1-activated expression of *OsSWEET11*, and bacterial virulence performance with a result of leaf blight formation in the susceptible rice variety.

### The Three Translocators Cooperate to Mediate PthXo1 Translocation

**FIGURE 5:**
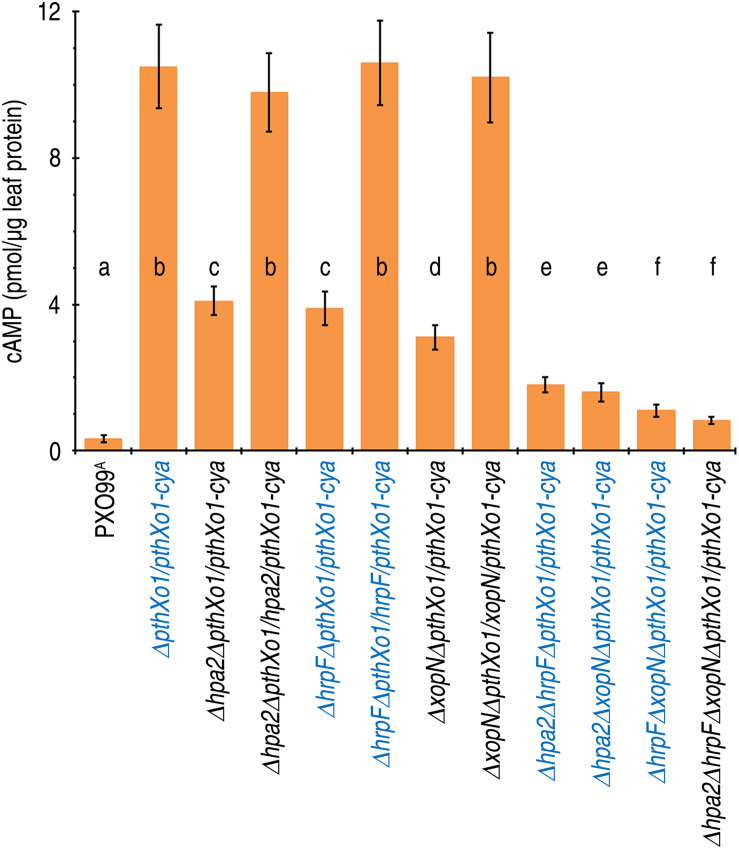
The cooperative effects of *hpa1*, *hpa2*, and *hrpF* on PthXo1 translocation levels quantified as cytoplasmic cAMP concentrations in rice leaves. Leaves of 14-day-old Nipponbare rice seedlings were inoculated by infiltration with bacterial suspensions of PXO99^A^ and *hpa1*, *hpa2*, and *hrpF* single, double, and triple mutants, respectively. 12 h later, inoculated leaves were excised and used in measurements of cytoplasmic cAMP concentrations. Data shown are the mean values ± SDs. Different letters on bar graphs indicate significant differences of data obtained from the plants inoculated with the different bacterial strains (ANOVA and Duncan’s test, *P* < 0.001, *n* = 3 independent experiment each involving 30 leaves of 15 plants).

To elucidate whether Hpa2, HrpF, and XopN synergize in mediating PthXo1 translocation, we performed parallel experiments to compare the single, double, and triple gene-knockout PXO99^A^ mutants with PXO99^A^ and Δ*pthXo1/pthXo1-cya* in terms of PthXo1 translocation levels. Leaves of 14-day-old Nipponbare seedlings were inoculated by leaf infiltration with every bacterial suspension of the specific *Xoo* strains, in which PXO99^A^ was used as a reference of background reading and Δ*pthXo1/pthXo1-cya* was assumed to provide the detectable maximal level of PthXo1 translocation. Based on cytoplasmic cAMP concentrations in leaves 12 hpi, PthXo1-Cya translocation incurred significant (*P* < 0.01) quantitative reductions in leaves of the seedlings inoculated with the single mutant Δ*hpa2ΔpthXo1/pthXo1-cya*, Δ*hrpFΔpthXo1/pthXo1-cya*, or Δ*XopNΔpthXo1/pthXo1-cya* compared with Δ*pthXo1/pthXo1-cya* (Figure 5). Further decreases in quantities of translocated PthXo1-Cya were found in the plants inoculated with the double and triple gene-knockout mutants (Figure 5). On average, 61, 63, and 71% reductions in PthXo1-Cya translocation levels were caused by deleting the single genes *hpa1*, *hrpF*, and *xopN*, respectively, under background of Δ*pthXo1/pthXo1-cya*. Further reductions up to 27% and 30% were induced by double and triple gene knockout, respectively. Moreover, the triple mutant Δ*hpa2ΔhrpFΔxopNΔpthXo1/pthXo1-cya* had the lowest amount of PthXo1-Cya translocation determined as cAMP content of 0.83 pmol/μg leaf protein, being 0.7% of the amount (cAMP 10.50 pmol/μg leaf protein) provided by Δ*pthXo1/pthXo1-cya*. Clearly, the concomitant presence of Hpa2, HrpF, and XopN is essential for the maximal translocation of PthoXo1, suggesting that the three translocators cooperate to mediate PthXol translocation from *Xoo* bacteria into rice cells.

### The Three Translocators Move Into Rice Cells Wherein Only XopN Suppresses Defense Responses

**FIGURE 6:**
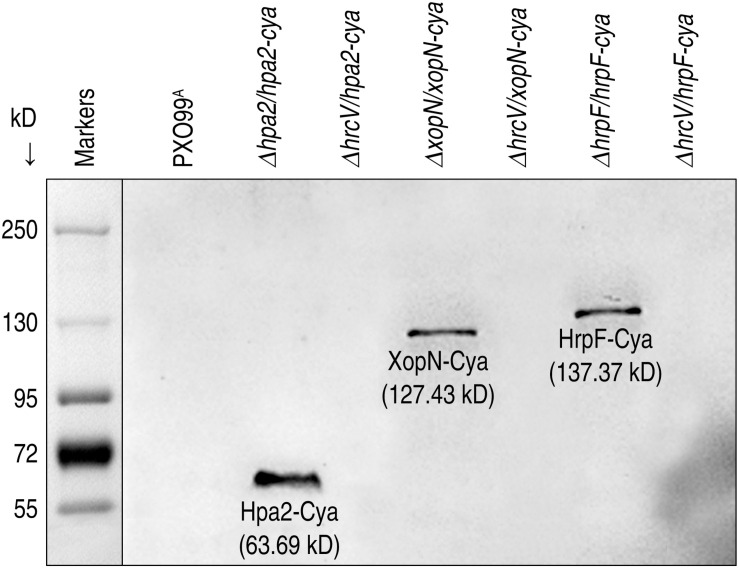
Hpa2, HrpF, and XopN translocation. Each of the three proteins was fused to a Cya sequence and introduced in the fusion form into bacteria of the PXO99^A^ mutants. Bacterial cultures of PXO99^A^ and the mutants were used to isolate soluble proteins. Protein samples were analyzed by immunoblotting with the specific antibody against Cya.

**FIGURE 7:**
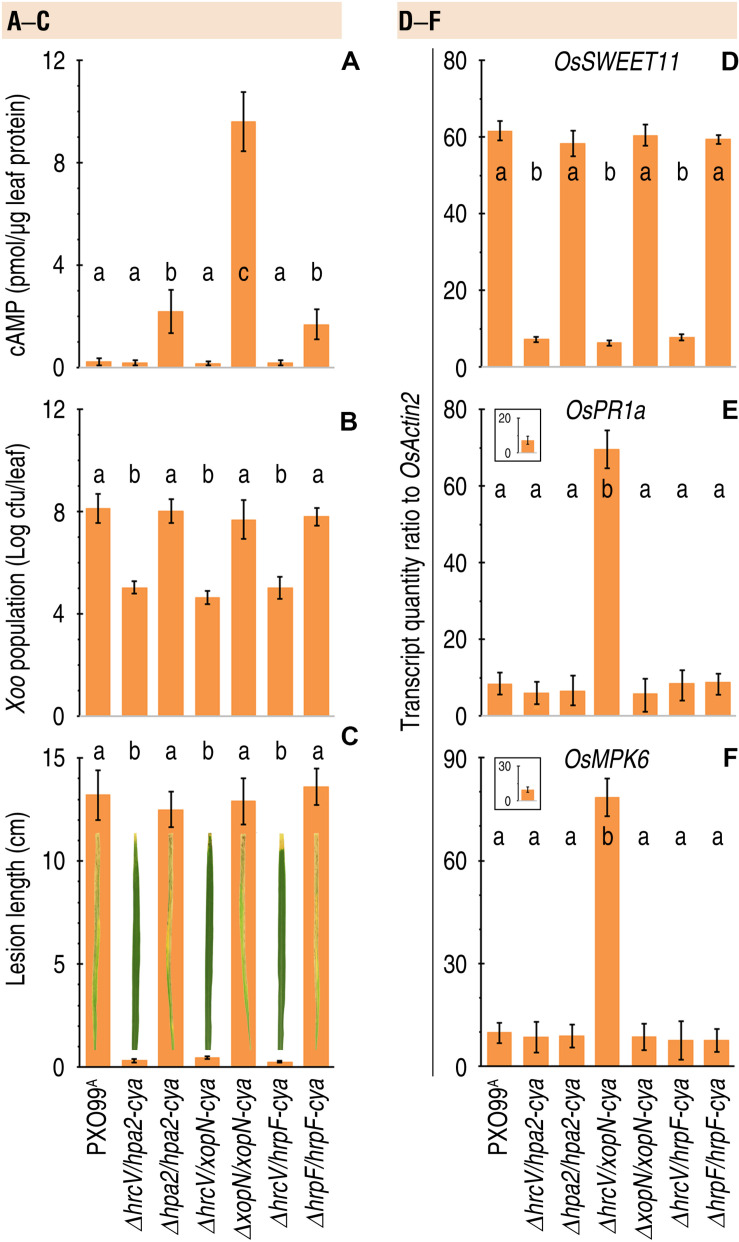
The coincident effects of Hpa2, HrpF, and XopN translocation on bacterial virulence and rice defense responses. **(A–F)** Leaves of 14-day-old Nipponbare rice seedlings were inoculated by infiltration with bacterial suspensions of PXO99^A^ and the complementing strains with and without the *hrcV* gene, respectively. Inoculated leaves were excised and used in the different assays. Quantitative data are given as the means ± SDs. Different letters on bar graphs indicate significant differences of data obtained from the experiments performed on the different bacterial strains (ANOVA and Duncan’s test, *P* < 0.001, *n* = 3 independent experiment each involving 30 leaves of 15 plants). **(A)** Translocation levels of the Cya-fused proteins shown as cytoplasmic cAMP concentrations in leaves 12 hpi. **(B)** Bacterial population in leaves 3 dpi. **(C)** Leaf blight symptoms and severity qualification at 7 dpi. **(D–F)** Rice gene expression in leaves 48 hpi. Gene expression was analyzed by RT-qPCR using the constitutively expressed *OsActin2* gene as a reference.

It is intriguing to know whether T3 translocators also undergo translocation as do T3 effectors. It is especially intriguing to know whether T3 translocators have additional pathological functions except for effector translocation. We addressed both questions by analyzing the correlation of bacterial virulence with secretion and translocation of the translocators from pertinent *Xoo* strains, including Δ*hrcV/hpa2-cya*, Δ*hpa2/hpa2-cya*, Δ*hrcV/xopN-cya*, Δ*xopN/xopN-cya*, Δ*hrcV/hrpF-cya*, and Δ*hrpF/hrpF-cya* tested in comparison with PXO99^A^. By protein secretion analysis, we confirmed that Hap2-Cya, HrpF-Cya, and XopN-Cya were secreted by the bacterial T3 pathway with the requirement for HrcV (Figure 6). We further determined that Hap2-Cya, HrpF-Cya, and XopN-Cya underwent translocation from bacteria into rice cells (Figure 7A). In the assay, 30-day-old Nipponbare rice seedlings were inoculated by leaf-top clipping with clinical scissors dipped in every bacterial suspension of the pertinent *Xoo* strains. Based on cytoplasmic cAMP concentrations in inoculated rice leaves measured at 12 hpi, the three translocators were translocated by the bacteria whose *hrcV* gene was reserved and were translocated at different extents (Figure 7A). While Hap2-Cya and HrpF-Cya had small amounts of translocation (2.2 and 1.7 pmol cAMP per μg leaf protein, respectively), XopN-Cya was translocated at a significantly (*P* < 0.001) increased concentration (9.6 pmol cAMP per μg leaf protein), scored as 3.5-fold higher than Hpa1 and 4.6-fold higher than HrpF. By contrast, the three translocators were little translocated from the Δ*hrcV/hpa2-cya*, Δ*hpa2/hpa2-cya*, and Δ*hrcV/xopN-cya* mutants generated by gene deletion and recombination under PXO99^A^ background (Figure 7A). Meanwhile, the *hrcV* gene knockout highly arrested bacterial virulence, significantly (*P* < 0.001) decreased bacterial populations in leaves as determined at 3 dpi (Figure 7B), and significantly (*P* < 0.001) reduced lesion length of leaf blight as observed at 12 dpi (Figure 7C). When *hrcV* is present, however, Δ*xopN/xopN-cya*, Δ*hpa2/hpa2-cya*, and Δ*hrpF/hrpF-cya* displayed similar levels of virulence, multiplied equally well in leaves (Figure 7B), and caused leaf blight with similar severities (Figure 7C). Furthermore, *OsSWEET11* was expressed equally well in leaves pf plants inoculated with Δ*xopN/xopN-cya*, Δ*hrcV/hrpF-cya*, and Δ*hrpF/hrpF-cya*, respectively, (Figure 7D). In other words, levels of *OsSWEET11* expression were similar (Figure 7D) although translocation levels of Hpa2-Cya and HrpF-Cya were small but the translocation amount of XopN-Cya was significantly (*P* < 0.001) higher (Figure 7A). Based on these results, we assumed that XopN might have more pathological roles than Hpa2 and HrpF in addition to the common function in mediating the bacterial effector translocation.

To validate this hypothesis, we elucidated whether defense responses occur in rice plants following translocation of the bacterial translocators. Considerable parts of plant defense responses are essentially subject to the ETI (effector-triggered immunity) and PTI (microbial pattern-triggered immunity) pathways (Kanyuka and Rudd, 2019). Therefore, we chose to analyze the foliar expression of *OsPR1a* and *OsMPK6* genes, which are molecular markers of the ETI and PTI pathways, respectively, (Zhang et al., 2019b). In the assay, 30-day-old Nipponbare rice plants were divided into two groups. In the first group used as defense response gene expression control, plants remained free from inoculation. In the second group, plants were inoculated by leaf clipping with clinical scissors dipped separately in bacterial suspensions of *Xoo* strains PXO99^A^, Δ*hrcV/hpa2-cya*, Δ*hpa2/hpa2-cya*, Δ*hrcV/xopN-cya*, Δ*xopN/xopN-cya*, Δ*hrcV/hrpF-cya*, and Δ*hrpF/hrpF-cya*. Subsequently, relative levels of *OsPR1a* and *OsMPK6* expression in inoculated leaves were analyzed at 48 hpi. Low basal levels of *OsPR1a* (Figure 7E inset) and *OsMPK6* (Figure 7F inset) expression were detected in leaves of plants that were not inoculated. In comparison, expression levels of both *OsPR1a* (Figure 7E) and *OsMPK6* (Figure 7F) were highly increased in, and only in, the plants inoculated with the recombinant bacterial strain Δ*xopN/xopN-cya*. By contrast, the expression of *OsPR1a* (Figure 7E), and *OsMPK6* (Figure 7F) were not evidently induced in the plants inoculated with PXO99^A^, Δ*hrcV/hpa2-cya*, Δ*hpa2/hpa2-cya*, Δ*hrcV/xopN-cya*, Δ*xopN/xopN-cya*, Δ*hrcV/hrpF-cya*, and Δ*hrpF/hrpF-cya*, respectively. Instead, transcripts of both defense response genes remained around the basal levels in the plants following inoculation with any of the bacterial strains in which a functional XopN was present (Figures 7E,F main graphs vs. insets). Based on these results, we propose that XopN present in PXO99^A^ acts to suppress defense responses in plants of the disease-susceptible rice variety under infection, but Hpa1 and HrpF do not have such a repressive effect on rice defense responses against *Xoo* infection.

## Discussion

Although the T3 translocon of eukaryote-pathogenic Gram-negative bacteria has been extensively studied in the past 20 years (Blocker et al., 2001; Büttner et al., 2002; Zhang et al., 2019a), characterization of the molecular mechanism that governs bacterial effector translocation is merely in the initial stage (Li et al., 2019; Picking and Barta, 2019). By browsing literatures (Table 1), we find that there was no study to demonstrate the composition of T3 translocon in different species of plant-pathogenic bacteria while infecting their host plants (Büttner, 2012; Ji and Dong, 2015b; Scheibner et al., 2017; Zhang et al., 2019a). Furthermore, in seven of eight previous reports that we have scrutinized with appreciation, bacterial effector translocation was measured by experiments performed on tobacco, the common non-host plant of almost all plant-pathogenic microbes, rather than host plants of the bacteria (Table 1). Evidently, looking for the composition of T3 translocon and demonstrating the functional connection between effector translocation and bacterial virulence are quite necessary especially for the study of plant-pathogenic bacteria.

**TABLE 1:** T3 translocators identified to date in plant-pathogenic bacteria.

Translocators	Characteristics	Bacteria	Effectors translocated	Plants	References
Hpa1	Hydrophilic	*Xanthomonas oryzae* pv. *oryzae* (*Xoo*)	PthXo1	Rice	Li et al., 2019
			AvrXa10	Rice	Wang et al., 2018
Hpa2	Hydrophilic	*X. oryzae* pv. *oryzae* (*Xoc*)	PthXo1	Rice	This study
			XopN	Rice	This study
		*Xoc*	AvrBs3	Tobacco	Li et al., 2011
HrpF	Hydrophobic	*X. campestris* pv. *vesicatoria*	AvrBs3	Pepper	Büttner et al., 2002
		*Xoo*	PthXo1, XopN	Rice	This study
		*Xoc*	AvrBs3	Tobacco	
HrpN_Ea_	Hydrophilic	*Erwinia amylovora*	DspA/E	Tobacco	Bocsanczy et al., 2008
HrpK1, HrpZ1, HrpW1, HopAK1	Hydrophilic	*Pseudomonas syringae* pv. *tomato*	AvrPto	Tobacco	Kvitko et al., 2007
PopA	Hydrophilic	*Ralstonia solanacearum*	AvrA	Tobacco	Racape et al., 2005
PopF1, PopF2	Hydrophobic	*Ralstonia solanacearum*	AvrA	Tobacco	Meyer et al., 2006

We have identified Hpa2, HrpF, and XopN as concomitant translocators of the *Xoo* TALE PthXo1, which was well studied with respect to its function in determining virulence of the standard *Xoo* strain PXO99^A^ on the susceptible variety Nipponbare (Yang et al., 2006; Wang et al., 2018; Bian et al., 2019; Li et al., 2019; Zhang et al., 2019b). Evidence that demonstrates the translocator role has been shown as quantitative changes of PthXo1 translocation in Nipponbare plants inoculated with the WT and genetically modified POX99^A^ strains in the presence and absence of Hpa2, HrpF, and XopN, respectively. T3 translocators share the common functional characteristic of requirement for the translocation, rather than secretion, of bacterial effectors (Yang, 2017). In agreement with this criterion, Hpa2, HrpF, and XopN regulate the virulent role of PthXo1 by mediating its translocation from bacteria into rice cells, but the three translocators are not required for PthXo1 secretion through the bacterial T3 pathway. Shortly before this study, we have already characterized Hpa1 as a translocator of *Xoo* TALEs PthXo1 and AvrXa10 trafficking from the bacteria into rice cells, wherein PthXo1 plays a virulent role, but AvrXa10 executes its avirulent function (Wang et al., 2018; Bian et al., 2019). Therefore, *Xoo* possesses at least four T3-translocators that function concomitantly to facilitate effector translocation.

Our results detailed in this article highlight the classical question regarding the functional coordination of different components in the bacterial T3-translocator repertoire (Mueller et al., 2008; Büttner, 2012; Choi et al., 2013; Ji and Dong, 2015b). Multiple (known 3 or 4) T3-translocators present in an infecting community (a single species, subspecies, pathovar, or serovar) of eukaryote-pathogenic bacteria show to have overlapping and redundant functions (Kvitko et al., 2007; Mueller et al., 2008; Ji and Dong, 2015b). In *Pst*, for example, four harpins (HrpK1, HrpZ1, HrpW1, and HopAK1) serve as semiredudant concomitant translocators of the TALE AvrPto (Kvitko et al., 2007). Deleting any of the *hrpK1*, *hrpZ1*, *hrpW1*, and *hopAK1* genes from the *Pst* genome causes a substantial reduction in AvrPto-Cya translocation, which is further diminished, but not eliminated, by the quadruple harpin gene polymutation (Kvitko et al., 2007). We show that Hpa2, HrpF, and XopN display a similar mode of functional redundancy. Deleting any of the *hpa1*, *hrpF*, and *xopN* genes from the PXO99^A^ genome causes a substantial reduction in PthXo1 translocation, which is further diminished, but not eliminated, by the triple gene polymutation. The involved mechanism remains mysterious.

We propose that the molecular mechanism that governs the functional redundancy of multiple translocators in an infecting bacterial community is possibly related to the hypothetic regulatory modes of bacterial T3 effector translocation in the translocon-dependent or -independent manner. The translocon-dependent model emphasizes molecular interactions between bacterial T3 translocators and molecular interactions of T3 translocators with their receptors (Büttner and Bonas, 2002; Büttner et al., 2008; Büttner, 2012; Ji and Dong, 2015b), either lipids (Haapalainen et al., 2011; Li et al., 2011), or proteins (Oh and Beer, 2007; Li et al., 2015; Adam et al., 2017) situated in the eukaryotic PMs. The translocon-independent model hypothesizes that bacterial T3 effectors move into eukaryotic host cells by two routes (Zhang et al., 2019a). In the first, bacterial effectors characteristic of cell-penetrating peptide (RPP) make pore in the target PM (Scharnert et al., 2013; Rüter and Schmidt, 2017). The second route is the effector endocytosis through direct interaction with receptors located in eukaryotic PMs (Domingues et al., 2016). Such a molecular interaction may trigger the membrane trafficking mechanism (Allgood and Neunuebel, 2018) either by endoplasmic reticulum or vesicles (Wudick et al., 2015), providing a potential scheme for bacterial effector endocytosis (Cybulsky, 2017; Obacz et al., 2017).

Because PthXo1 does not belong to RPP, its translocation is not likely to use the pore formation mechanism. Instead, PthXo1 translocation needs typical T3 translocators Hpa1 (Wang et al., 2018), Hpa2 (Li et al., 2011), and HrpF (Büttner et al., 2002). Both Hpa1 (Chen et al., 2008a) and Hpa2 (Li et al., 2011) belong to hydrophilic proteins previously defined as T3 accessory proteins (Zhu et al., 2000; Choi et al., 2013). HrpF is an atypical hydrophobic protein, contains a hydrophobic group in the C-terminal sequence, but lacks hydrophilic groups in the N-terminal sequence (Büttner et al., 2002). HrpF is the T3 translocator first discovered in plant-pathogenic bacteria and is considered an essential component of the T3 translocon (Büttner et al., 2002; Scheibner et al., 2017). Therefore, it is very likely that PthXo1 is translocated in the translocon-dependent manner when the infecting bacteria can secrete all translocators required. Based on the functional redundancy of the concomitant translocators, we hypothesize that the translocon may deploy an adjustable mechanism to alter its functional levels according to variations in the translocator compositions. Such an adjustable mechanism is deployed to ensure effector translocation in the absence of any translocators, preventing the effector translocation from termination by loss-of-function mutation of any translocators. This hypothesis needs to be validated.

It is intriguing that T3 translocators also move into plant cells as do effectors as found so far in studies which have determined whether translocators undergo translocation (Kvitko et al., 2007; Mo et al., this study). In the current model, nevertheless, T3 translocators do not necessarily need to be translocated. Instead, they can fulfill their functions in mediating effector translocation if they interact each other according to the hierarchical sequence of secretion and meanwhile interact with the corresponding receptors situated in eukaryotic PMs (Büttner, 2012; Ji and Dong, 2015b). However, T3 translocators authentically undergo the transkingdom transport from bacteria into the cytosol of plant cells (Kvitko et al., 2007; Mo et al., this study). In this case, the translocators may execute more functions than mediating bacterial effector translocation. This notion has been partially validated by our finding about the dual functions of XopN in mediating PthXo1 translocation and in suppressing plant defense responses following the translocation by itself. At present, however, we do not have experimental evidence to show what responses, in addition to PthXo1 translocation, could be further induced by Hpa1, Hpa2, and HrpF translocated into rice cells. By contrast, it is convincing to propose that XopN is not only a T3 effector as previously defined, but also a T3 translocator as now appreciated. In conjecture, XopN executes the dual functions possibly relate to its trafficking route toward plant PMs (Kim et al., 2009). On the one hand, this subcellular localization satisfies the requirement for a T3 translocator to function by association with a specific receptor at eukaryotic PMs (Büttner, 2012; Ji and Dong, 2015b). On the other hand, following localization at the PM-cytoplasm interface, XopN interacts with TARK1 (Kim et al., 2009), and 14-3-3 proteins (Taylor et al., 2012) to induce suppression of the PTI signaling pathway (Taylor et al., 2012). Whether a similar signaling mechanism is employed by *Xoo* XopN translocated into rice cells remains to be studied.

Taken together, our findings in this study and previous works (Wang et al., 2018; Bian et al., 2019; Li et al., 2019; Zhang et al., 2019b) demonstrate that Hpa1, Hpa2, HrpF, and XopN are concomitant translocators of the TALE PthXo1 in the standard *Xoo* strain PXO99^A^ while infecting the susceptible rice variety Nipponbare. We further show that in addition to mediate PthXo1 translocation, the three translocators themselves also move into rice cells, wherein XopN has a high quantity and induces suppression of rice defense responses. In the initial motivation, we did not intend to analyze whether the translocators function as specialists for PthXo1 only or generalists for more effectors. Instead, we have paid constant attentions to integral components of the T3 translocon and the assumed intricate process of the translocon assembly (Picking and Barta, 2019) particularly in plant-pathogenic bacteria (Ji and Dong, 2015b; Zhang et al., 2019a). Characterization of the translocon assembly has been a highly challengeable work for the scientific community. Identifying T3 translocators in *Xoo* is an indispensable step toward dissecting the bacterial T3 translocon assembly in the future.

## Materials and Methods

### Plant Growth Condition

Nipponbare seeds were initially provided by our colleague Professor Hongsheng Zhang (College of Agronomy, Nanjing Agricultural University) and were then reproduced and maintained in this lab. Seeds were germinated on filter papers immersed in sterile water in Petri dishes. After 3–5 days, the germinal seedlings were moved into 12-L pots (2–5 plants per pot) filled with a substrate containing peat, sand and vermiculite (1:1:1 v/v). Seeds were incubated and the plants were grown in environment-controlled chambers under 26 ± 1°C, 12-h light at 250 ± 50 μmol quanta/m^2^/s and a relative humidity of 85%.

### Bacterial Strains and Plasmid Vectors

Bacterial strains and plasmid vectors used and created in this study and information on antibiotic resistance and other elements are provided in Supplementary Table 1. *Escherichia coli* was grown at 37°C in Luria-Bertani broth (LB) or on LB agar (LA) plates with the appropriate antibiotics. *Xoo* strains were cultured at 28°C on nutrient broth (NB) or NA agar (NA) medium (Li et al., 2011). Bacteria were cultured on medium supplemented with 50 μg/mL ampicillin, 100 μg/mL spectinomycin, or 50 μg/mL kanamycin.

### Bacterial Mutant Generation and Complementation

Gene deletion from and backfill to the PXO99^A^ genome were performed by using the unmarked deletion method (Song and Yang, 2010). The *hrcV* and *pthXo1* gene-knockout mutants and complementing strains were generated previously in our lab (Wang et al., 2018; Li et al., 2019). To create *hpa2*-, *hrpF*-, and *xopN*-knockout mutants, upstream and downstream flanking partial sequence fragments of each gene were amplified from the PXO99^A^ genomic DNA and were connected by overlapped fusion-PCR using specific primers (Supplementary Table 2). Every PCR product was confirmed by sequencing and then cloned into the vector pK18*sacB* by digestion with the corresponding restriction enzymes (Supplementary Table 2) and ligation with T4 ligase (Thermo Scientific, Walsham, Massachusetts, United States). Every recombinant vector was introduced into PXO99^A^ cells by electroporation, followed by single-colony selection on kanamycin-containing and sugar-absent NA plates. Colonies from single crossovers were transferred to NB broth, grown at 28°C for 12 h and then transferred onto plates containing NA and 10% sucrose. Sucrose-resistant colonies were replica streaked onto NA plates with and without kanamycin supplementation. Colonies resulting from double crossover events were selected based on kanamycin-negative and sucrose-positive traits, and unmarked mutants (Δ*hpa2*, Δ*hrpF*, and Δ*xopN*) were confirmed by PCR amplification of *hpa2*, *hrpF*, and *xopN*, respectively. To create double and triple mutants, the recombinant vectors pK18*sacB*:Δ*hpa2*, pK18*sacB*:Δ*hrpF*, and pK18*sacB*:Δ*xopN* were transferred correspondingly into one of the Δ*hpa2*, Δ*hrpF*, and Δ*xopN* mutants or double mutants generated in the transformation process.

Different tags were attached to the 3’-terminus of the *hpa2*, *hrpF*, *pthXo1*, or *xopN* sequence in the pZW*pthXo1* plasmid vectors (Wang et al., 2018). To create a *cya*-fused gene, a 1218-bp *cya* fragment encoding amino acids 2 to 406 of the Cya protein was amplified from plasmid pMS107 and prefixed with the last 51-bp region of *hpa2*, *hrpF*, *xopN*, or *pthXo1* that contained a *Sac*I recognition site (Supplementary Table 2). The recombinant sequence was inserted into pZW*pthXo1* at the *Sac*I site. Every recombinant vector was linearized with *Hin*dIII and cloned into the pHM1 vector for genetic complementation. The double complementary vectors, for example that for *hpa1pthXo1*, were constructed using two steps. First, the *hpa1* sequence that was linked its own promoter was cloned into pHM1 between the *Pst*I and *Kpn*I sites. Second, pZW*pthXo1* was linearized using *Hin*dIII and inserted into the *Hin*dIII site of pHM1*hpa1*. Complementation or transformation was performed by electroporation. Similar procedures were used to generate double complementation of other gene combinations.

### Bacterial Virulence Assessments

Overnight *Xoo* NA cultures were washed twice and resuspended in sterile water to generate inoculum suspensions with an optical density of OD_600_ = 0.3. A bacterial suspension was inoculated on leaves of 14-day-old rice seedlings by infiltration with needleless syringes at 3 sites per leaf. Alternatively, a bacterial suspension was inoculated on leaves of 30-day-old rice plants by the leaf-clipping method (Li et al., 2011). Symptoms were scored by photographing or measuring lesion lengths or areas. Bacterial growth in rice leaves was measured by harvesting 10 leaves for each group of plants inoculated with a single bacterial strain.

### Bacterial Protein Secretion Assays

Cya-containing PXO99^A^ strains were grown in NB broth at 28°C with the appropriate antibiotics to logarithmic phase. Bacterial cells were harvested by centrifugation. The precipitated bacterial pellet was washed twice with sterile water and resuspended in 100 mL of type III-inducing XOM2 liquid media (Tsuge et al., 2002) to an of OD_600_ = 0.6. This XOM2 suspension was supplied with the appropriate antibiotics and incubated in a 28°C shaker at 220 *rpm* for 16 h. XOM2 cultures were then separated into cell pellet and supernatant fractions by centrifugation. The proteins in the pellet and supernatant were extracted by sonication and by precipitation with 12.5% trichloroacetic acid, respectively, (Li et al., 2011). Proteins were separated by 6% SDS-PAGE and transferred to Immobilon-P membranes (Millipore) for immunoblotting analyses using a Cya antibody (Santa Cruz) or β-lactamase antibody (Abcam). The ampicillin resistance protein β-lactamase protein is encoded by the pZW*tal-cya* vector (Supplementary Table 2), remains cell-bound unless non-specific cell leakage occurred, and was used as a control for non-specific cell lysis. Protein blots were incubated with the specific antibody and hybridized to horseradish peroxidase-conjugated goat antimouse immunoglobulin G from the BeyoECL Plus kit (Beyotime).

### Cya Reporting

The Cya reporter is an accurate and complete roster of effector proteins translocated by pathogenic bacteria into host cells, offering a highly sensitive and robust assay for monitoring the translocation of the effectors (Chakravarthy et al., 2017). An effector is fused to the calmodulin-dependent adenylate-cyclase domain of CyaA. The increase in cAMP concentration in plant cells is then measured with an enzyme-linked immunosorbent assay kit. This assay was performed on 14- or 30-day-old rice seedlings inoculated with Cya-related *Xoo* strains. Bacterial suspensions were prepared from NA cultures and adjusted to an OD_600_ = 0.3. Each suspension was infiltrated into intercellular spaces of expanded leaves at three sites per leaf. At the designed time points, inoculated leaves were excised from inoculated leaves, weighted, and then frozen in liquid nitrogen in a mortar and ground with a ceramic pestle to a fine powder. The leaf powder was suspended in 350 μl of buffer A provided in the cAMP ELISA detection kit (GenScript Biotech Corp, Nanjing, China), followed by brief centrifugation at 10,000 *g* for 10 min. The supernatant was analyzed as per the manufacturer’s instruction book to determine intracellular cAMP concentrations. Total proteins in each sample for normalization were quantified by using a BCA protein assay kit (TransGen Biotech, Beijing, China).

### Rice Gene Expression Analysis

Total RNA was isolated from leaves or protoplasts by using TRIzol (Invitrogen) and treated with DNase I (Invitrogen) to remove DNA. Northern blotting and RT-qPCR were performed as previously described (Li et al., 2019). The constitutively expressed *OsActin2* gene was used as a reference. RT-qPCR analyses always employed specific primers (Supplementary Table 2) and included temple-absent controls. Relative expression level of a gene tested was quantified as the transcript quantity ratio to *OsActin2*.

### Data Treatment

All experiments were repeated at least three times with similar results as provided. Quantitative data were analyzed by using the commercial IBM SPSS19.0 software package (Shi, 2012) installed in the registered office computers. Homogeneity-of-variance in data was determined by Levene test. Formal distribution pattern of the data was confirmed by Kolmogorov-Smirnov test and P-P Plots. Analysis of variance (ANOVA) was performed along with Duncan’s test for multiple comparisons (Li et al., 2014) of data from at least three independent experiments each involving three repetitions at minimum unless specified elsewhere in the case that a leaf was treated as a statistical unit.

## Data Availability Statement

The raw data supporting the conclusions of this article will be made available by the authors, without undue reservation, to any qualified researcher.

## Author Contributions

XM and LZ designed and performed the experiments and wrote the manuscript. YL, XW, JB, and KL performed the experiments. SZ analyzed the data and commented on the manuscript. LC supervised the research and commented on the manuscript. HD conceived the research, designed the experiments, and wrote and finalized the manuscript.

## Conflict of Interest

HD, LZ, and LC are the inventors on a provisional patent application 201911394862.6 that covers an Xoo T3 translocator. The remaining authors declare that the research was conducted in the absence of any commercial or financial relationships that could be construed as a potential conflict of interest.
