# Supplementary material for: Three Proteins (Hpa2, HrpF and XopN) Are Concomitant Type III Translocators in Bacterial Blight Pathogen of Rice
Source: Front Microbiol. 2020 Jul 22;11:1601. doi: 10.3389/fmicb.2020.01601 (PMC7390958; doi:10.3389/fmicb.2020.01601)
Supplement: Supplementary file 1 [file Data_Sheet_1.docx]

Three Proteins (Hpa2, HrpF and XopN) Are Concomitant Type III Translocators in Bacterial Blight Pathogen of Rice

**Xuyan Mo, Liyuan Zhang, Yan Liu, Xuan Wang, Jiaqi Bai, Kai Lu, Shenshen Zou, Lei Chen and Hansong Dong**

**Supplementary Material**

**
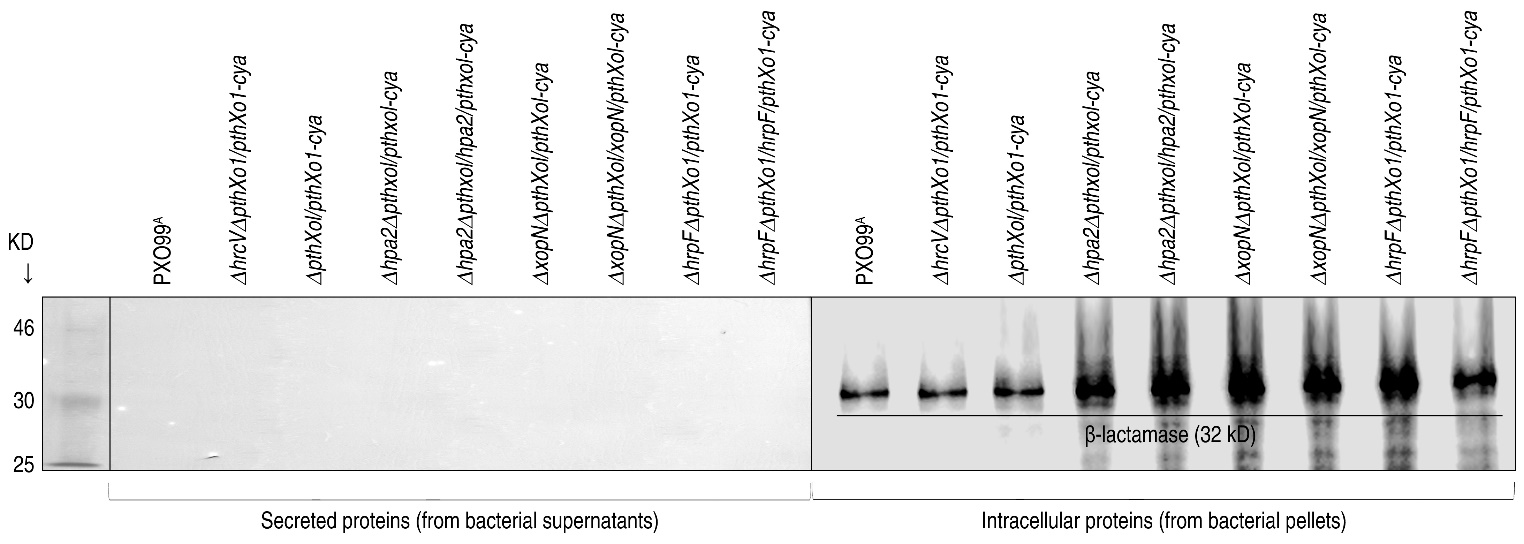
**

**SUPPLEMENTAL FIGURE 1 |** Immunoblotting of bacterial proteins hybridized in blots with the specific antibody against β-lactamase that is a cytosolic protein used as a lysis control.

**
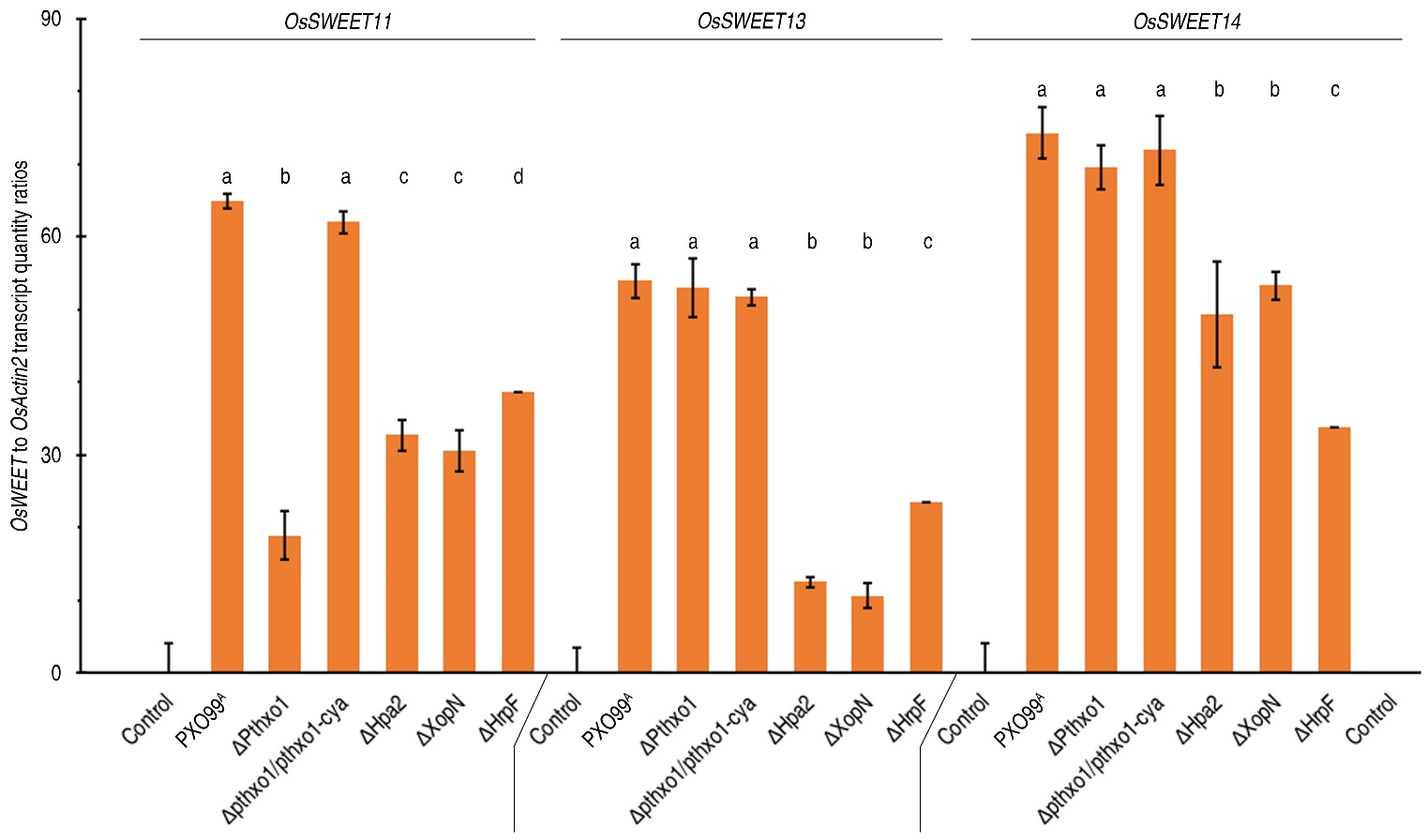
**

**SUPPLEMENTAL FIGURE 2 |** Expression of three *OsSWEET* genes in leaves of rice plants inoculated with the *Xoo* strain PXO99^A^ and the genetically recombinant strains, respectively. Leave of 30-day-old Nipponbare plants were inoculated by leaf top clipping with clinical scissors dipped in a bacterial suspension of every strain. After 24 hours, inoculated leaves were excised and used in RNA extraction. Isolated leaf RNAs were analyzed by RT-qPCR using the constitutively expressed *OsActin2* gene as a reference. “Control” was the reaction without supply of leaf RANs. Data shown are mean values ± standard deviation estimates. Different letters on bar graphs indicate significant differences of data obtained from the experiments performed on the different bacterial strains (ANOVA and Duncan’s test, *P* < 0.001).

**SUPPLEMENTAL TABLE 1 |** Bacterial strains and plasmids used in this study

| Strains or plasmids | Relevant characteristics | Source/reference |
| --- | --- | --- |
| **Strains** |  |  |
| *Escherichia coli* |  |  |
| DH5α | F^–^ 80d*lacZ* M15(*lacZYA-argF*) U169 *endA1 deo*R *rec*A1 *hsd*R17(r_K_^–^ m_K_^+^) *pho*A *sup*E44 λ^–^ *thi-l gyr*A96 *rel*A1 | This lab |
| BL21(DE3) | F^–^ *omp*T *hsd*SB (*r_B_^–^ m_B_^–^*) *gal dcm* (DE3) | Novagen |
| *X. oryzae* pv. *oryzae* |  |  |
| PXO99^A^ | Philippine race 6 (PXO99) modified to carry an azacytidine-resistant gene, virulent to the rice cultivar Nipponbare | This lab |
| PXO99^A^ mutants |  |  |
| *Δhpa2*  *ΔxopN*  *ΔhrpF*  *ΔpthXo1*  *Δhpa2ΔpthXo1*  *ΔxopNΔpthXo1*  *ΔhrpFΔpthXo1*  *ΔhrcV*  *Δhpa2/hpa2*  *ΔxopN/xopN*  *ΔhrpF/hrpF*  *ΔpthXo1/pthXo1*  *Δhpa2ΔpthXo1/hpa2/pthXo1*  *ΔxopNΔpthXo1/xopN/pthXo1*  *ΔhrpFΔpthXo1/hrpF/pthXo1*  *ΔpthXo1/pthXo1-cya*  *Δhpa2ΔpthXo1/pthXo1-cya*  *ΔxopNΔpthXo1/pthXo1-cya*  *ΔhrpFΔpthXo1/pthXo1-cya*  *Δhpa2ΔpthXo1/hpa2/pthXo1-cya*  *ΔxopNΔpthXo1/xopN/pthXo1-cya*  *ΔhrpFΔpthXo1/hrpF/pthXo1-cya*  *ΔhrcVΔpthXo1/pthXo1-cya*  *Δhpa2/hpa2-cya*  *ΔxopN/xopN-cya*  *ΔhrpF/hrpF-cya*  *ΔhrcVΔhpa2/hpa2-cya*  *ΔhrcVΔxopN/xopN-cya*  *ΔhrcVΔhrpF/hrpF-cya*  *Δhpa2ΔhrpFΔpthXo1/pthXo1-cya*  *Δhpa2ΔxopNΔpthXo1/pthXo1-cya*  *ΔhrpFΔxopNΔpthXo1/pthXo1-cya*  *Δhpa2ΔhrpFΔxopNΔpthXo1/pthXo1-cya* | *hpa2* unmarked mutant  *xopN* unmarked mutant  *hrpF* unmarked mutant  *pthXo1* unmarked mutant  *hpa2pthXo1* double unmarked mutant  *xopNpthXo1* double unmarked mutant  *hrpFpthXo1* double unmarked mutant  *hrcV* knock-out mutant  *hpa2* mutant complemented with *hpa2*  *xopN* mutant complemented with *xopN*  *hrpF* mutant complemented with *hrpF*  *pthXo1* mutant complemented with p*pthXo1*  *hpa2pthXo1* mutant complemented with *hpa2pthXo1*  *xopNpthXo1* mutant complemented *xopNpthXo1*  *hrpFpthXo1* mutant complemented with *hrpFpthXo1*  *pthXo1* mutant complemented with *pthXo1-cya*  *hpa2pthXo1* mutant complemented with *pthXo1-cya*  *xopNpthXo1* mutant complemented with *pthXo1-cya*  *xopNpthXo1* mutant complemented with *pthXo1-cya*  *hpa2pthXo1* mutant complemented with *hpa2pthXo1-cya*  *xopNpthXo1* mutant complemented with *xopNpthXo1-cya*  *hrpFpthXo1* mutant complemented with *hrpFpthXo1-cya*  *hrcVpthXo1* mutant transformed with *pthXo1-cya*  *hpa2* mutant complemented with *hpa2-cya*  *xopN* mutant complemented with pHM*xopN-cya*  *hrpF* mutant complemented with *hrpF-cya*  *hrcVhpa2* mutant transformed with *hpa2-cya*  *hrcVxopN* mutant transformed with *xopN-cya*  *hrcVhrpF* mutant transformed with *hrpF-cya*  *hpa2hrpFpthXo1* mutant transformed with *pthXo1-cya*  *hpa2xopNpthXo1* mutant transformed with *pthXo1-cya*  *hrpFxopNpthXo1* mutant transformed with *pthXo1-cya*  *hpa2hrpFxopNpthXo1* mutant transformed with *pthXo1-cya* | This study  This study  This study  This lab  This study  This study  This study  This lab  This study  This study  This study  This lab  This study  This study  This study  This lab  This study  This study  This study  This study  This study  This study  This lab  This study  This study  This study  This study  This study  This study  This study  This study  This study  This study |
| **Plasmids** |  |  |
| pK18*sacB*  pHM1  pZW*pthXo1*  pZW*pthXo1-cya*  pMD18-T simple  pMS107  pET30a (+)  pET41a (+) | Suicide vector derivative from pK18mobGII, *sacB^+^*, Km^R^  Broad-host range vector with pUC19 polylinker, Sp^R^  PthXo1 fused to *lacZ* promoter of pBluescript II KS(+)  Cya tag inserted in the *Sac* I site of pZW*pthXo1*  pUC *ori*, cloning vector, Amp^R^  pLC20H containing the PCR fragment of *cyaA* from nucleotide 4 to nucleotide 1221, Amp^R^  pBR322 origin, *T7* promoter His-tag, Km^R^  pBR322 origin, *T7* promoter His-tag, GST-tag, Km^R^ | This study  This study  Yang et al., 2006  This study  Takara  This lab  Novagen  Novagen |

**SUPPLEMENTARY TABLE 2 |** Information on genes tested and primers used in this study

| Gene (Locus number) | Primers / product size (bp) / subjects |
| --- | --- |
| *hpa2* ([ACD56759.1](https://www.ncbi.nlm.nih.gov/protein/188518814)) | 5’-GTGTTGCGTGCCATTGCCTG-3’, 5’-CTATTCACCAATCACACCAC-3’ / 327 / coding sequence cloning by PCR |
|  | 5’-CGTGACGATTCCTCTCTGAT-3’, 5’-CTATTCACCAATCACACCAC-3’ / 1238 / promoter + coding sequence |
|  | Upstream homologous arm:  5’-G**gaatcc**CCCCGATCAAACAGTAGAAG-3’ (*EcoR*I),  5’-ACGC**gtcgac**CCAAGGGTTGACGTGCTGAT-3’ (*Sal*I) / 524,  Downstream homologous arm:  5’-C**cccggg**GGCAAGTGATCCTGCAGGGA-3’ (*Sma*I),  5’-GC**tctaga**TACAAGGTCATTGCCGGCAC-3’ (*Xba*I) / 541 / deletion |
|  | 5’-CG**ggatcc**CGTGACGATTCCTCTCTGAT-3’ (*BamH*I),  5’-CG**ggatcc**CTATTCACCAATCACACCAC-3’ (*BamH*I) / 1238 / complemention |
| *xopN* ([ACD61453.1](https://www.ncbi.nlm.nih.gov/protein/188523508)) | 5’-TTGACCGATGCCGCCACT-3’, 5’-TTACGCCGGCGGCAGTGCCCGATCCTCCT-3’ / 2205 / coding sequence cloning by PCR |
|  | 5’-AGAACGCACGCTGGGTCAAC -3’, 5’-TTACGCCGGCGGCAGTGCCCGATCCTCCT-3’ / 3234 / promoter + coding sequence |
|  | Upstream homologous arm:  5’-G**gaatcc**ATGCGGCACCCGCT-3’ (*EcoR*I),  5’-ACGC**gtcgac**ATCTGGCTTGGCACA-3’ (*Sal*I) / 500,  Downstream homologous arm:  5’-ACGC**gtcgac**GGCTCACAATCGGAATCT-3’ (*Sal*I) ,  5’-GC**tctaga**ATGGCCGCTGCCGGGTAAT-3’ (*Xba*I) / 500 / deletion |
|  | 5’-CC**aagctt**AGAACGCACGCTGGGTCAAC-3’ (*Hind*III),  5’-CC**aagctt**TTACGCCGGCGGCAGTGCCCGATCCTCCT’ (*Hind*III) / 3234/ complemention |
| *hrpF* ([ACD56731.1](https://www.ncbi.nlm.nih.gov/protein/188518786)) | 5’-ATGTCGCTCAACATGCTTTC-3’, 5’-TTATCTGCGACGTATCCTGA-3’ / 2409 / coding sequence cloning by PCR |
|  | 5’-ATACACGAAGTTGGGCGCAT -3’,  5’-TTATCTGCGACGTATCCTGA-3’ / 4429 / promoter + coding sequence |
|  | Upstream homologous arm:  5’-G**gaatcc**TGAGGTATTCACTATCTGCG -3’ (*EcoR*I),  5’-ACGC**gtcgac**ATAGGCGGCCTCGCGAATGA-3’ (*Sal*I) / 510  Downstream homologous arm:  5’-ACGC**gtcgac**CGCGCTTACCACAGCGAGGTTGCGTTAG-3’ (*Sal*I),  5’-GC**tctaga**TACTGCGATGCGACTGCCAT-3’ (*Xba*I) / 520/ deletion |
|  | 5’-CC**aagctt**ATACACGAAGTTGGGCGCAT-3’ (*Hind*III),  5’-CC**aagctt**TTATCTGCGACGTATCCTGA-3’ (*Hind*III) / 4429 / complemention |
| *hpa2*:*cya* | 5’-GC**gagctc**taaacttatacgaaggtgctctgccaattctgtcattcgcgaggccgccta (*hpa2*)  GGGCAGCAATCGCATCAGGCT (*cya*)-3’ (*Sac*I),  5’-GC**gagctcaagctt**TTAGCTGTCATAGCCGGAATCC-3’ (*Sac*I-*Hind*III) / 1221 / complementation, secretion, and translocation (Cya reporting) |
| *xopN*:*cya* | 5’-GC**gagctc**gaggtcatcgtgcagatcggggaggaggatcgggcactgccgccggcgtaa (*xopN*)  GGGCAGCAATCGCATCAGGCT (*cya*)-3’ (*Sac*I),  5’-GC**gagctcaagctt**TTAGCTGTCATAGCCGGAATCC-3’ (*Sac*I-*Hind*III) / 1221 / complementation, secretion, and translocation |
| *hrpF*:*cya* | 5’-GC**gagctc**gagggagacgtgcggcagaatctgcgcaatgtcaggatacgtcgcagataa (*hrpF*)  GGGCAGCAATCGCATCAGGCT (*cya*)-3’ (*Sac*I),  5’-GC**gagctcaagctt**TTAGCTGTCATAGCCGGAATCC-3’ (*Sac*I-*Hind*III) / 1221 / complementation, secretion, and translocation |
| *pthXo1* (AY495676) | Upstream homologous arm:  5’-CG**ggatcc**CTATTCGGATTTCGTGCTGTAC-3’ (*BamH*I),  5’-CAAACTGCTATGCCCAGGCTTATCGTTGTGCTCGTCCCTTTTG-3’ / 513  Downstream homologous arm:  5’-CAAAAGGGACGAGCACAACGATAAGCCTGGGCATAGCAGTTTG-3’,  5’-GC**tctaga**ATCGTGGAATTCGCCAAGGAAT-3’ (*Xba*I) / 605 / deletion |
| *OsSWEET11* (AK070510) | 5’-TGGTTCTGCTACGGCCTCTT-3’, 5’-GGTACCAGAAGTAGAGCCCCATCT-3’ / 103 / real-time RT-PCR |
| *OsSWEET13* (AK242853) | 5’-GCCTGTCCCTGCAGCATC-3’, 5’-CTCCGTCGACTTGCTCTTGTAG-3’ / 122 / real-time RT-PCR |
| *OsSWEET14* (AK101913) | 5’-CATCGTGGTTCTTGGTTGGG-3’, 5’-GAGAACGGCATGAACTCCAC-3’ / 114 / real-time RT-PCR |
| *OsActin2* | 5’-CCCCTGAGGAGCACCCAGTTCTA-3’, 5’-CATACCCCTCGTAGATTGGCACAG-3’ / 219 / real-time RT-PCR |
